# At-Risk but Unseen: Overdose Risk Documentation in Opioid Substitution Therapy Outpatients.

**DOI:** 10.1192/bjo.2026.11533

**Published:** 2026-06-30

**Authors:** Mohammad Ali

**Affiliations:** Addictions Recovery Community (ARC-MK), Milton Keynes, United Kingdom

## Abstract

**Aims:**

This service evaluation aimed to assess how consistently overdose risk factors are recorded in routine outpatient clinical records, identify gaps in documentation, and explore patient characteristics associated with missing or incomplete risk assessments.

**Methods:**

A retrospective service evaluation was conducted in an addictions psychiatry outpatient clinic. Adults aged ≥18 years receiving methadone or buprenorphine during a six-month period were included. Patients were excluded if they had transferred care during this period or had incomplete medical records. Clinical notes and pharmacy records were reviewed to extract information on overdose risk factors, including polysubstance use, previous overdose, co-prescribed benzodiazepines, psychiatric comorbidities, and naloxone provision. Demographic information and substance use patterns were also collected. Data were analysed descriptively to summarise documentation rates, and exploratory comparisons were made between patients with and without recorded overdose risk factors

**Results:**

A total of 110 patients were included (methadone n=68, buprenorphine n=42). Overdose risk factors were documented in 61 patients (55%), leaving 45% without recorded risk assessment. Naloxone provision was documented in only 44 patients (40% of total), including 12 patients with known polysubstance use who had no recorded naloxone offer. Polysubstance use was present in 48 patients (44%), and these individuals were more likely to have risk factors documented (65% vs 48%), although a substantial proportion remained undocumented. Patients with co-occurring psychiatric diagnoses were also more likely to have documented overdose risk, but documentation consistency varied widely. The review demonstrated that systematic evaluation of overdose risk documentation is feasible within routine outpatient records and can identify gaps that may compromise patient safety.

**Conclusion:**

Overdose risk is inconsistently documented in OST outpatient settings, despite high prevalence of polysubstance use and psychiatric comorbidity. Gaps in documentation, including missed naloxone provision, highlight the need for structured protocols and staff training to ensure that high-risk patients are identified and receive appropriate harm reduction interventions. Implementing routine overdose risk assessments within clinical workflows is feasible and may improve safety, patient outcomes, and overall quality of care.

